# A New Punishment for Murder

**Published:** 1893-07

**Authors:** 


### "A NEW PUNISHMENT FOR MURDER."

Dr. W. A. Hammond does not approve of the death penalty, but recommends as a substitute *castration*. He thinks nearly every man would rather die than submit to the operation, and that castration would be infinitely more effective in preventing crime than the death penalty, either by hanging or electrocution. Dr. Hammond quotes a French writer, with evident approval, that the dignity of a man resides in his testicles. "Juries would be less squeamish about discerning guilt, in cases of doubtful testimony, if life-taking were not one of the consequences of finding a man guilty. The punishment would be continuous, and not momentary and intense, and in the continuance of a punitive award will be found its greatest effect. Castration changes the whole relations of the man, and, while the brand upon him would be as bad or worse than the mark upon Cain, he would be removed from many criminal tendencies. He cannot open his mouth without exposing what has happened to him; his facial expression becomes altered; he becomes effeminate and cowardly; he loses his appetite for alcoholic drink. In the case of a man who mutilated himself while delirious from alcohol, this act was the means of his reformation.

"Criminals thus changed might, in many instances, be made useful to society, much more so than dead men, even if it be the law that slays them. They could be made members of church choirs—the vocal qualifications of eunuchs are well known—safe typewriters, dry nurses, laundrymen, policemen, sailors, soldiers, legislators, reporters for mild society newspapers, and other places where originality and 'dash' are not requisite.

"The brutality of animal nature is reduced by emasculation," Dr. Hammond continues to argue. "The fierce ram becomes mild, and the night-roving tom-cat ceases his pugnacious raids. The eunuchs of the Orient are described by travelers as a mild and obedient type of of persons, generally trustworthy and fond of the care of young children.

"As a means of stopping the propagation of criminals, this form of punishment would be no less effective than hanging. If it had been employed for the last few hundred years, the number of criminal acts would have been many thousands less by this time, and the administration of justice would now have become much simpler and less expensive.

"This punishment, of course, cannot apply to women. They have become so accustomed to the removal of ovaries, and these organs as so much less essential to womanhood than the testicles are to manhood that it might be necessary to substitute imprisonment for life in their case."

In Delaware it is found that a certain class of crime is best treated by the whip, which is a degradation which to even the most depraved is a terror exceeding by far that of imprisonment at hard labor. We suggest that, before taking so grave a step as to make for men castration a substitute for the death penalty, that it be tried upon the wife-beater and that class of depraved so low in the scale of humanity that law and decency are not sufficient to protect society from their lust. Occasionally a man is sent to prison for rape, and in one or two States for wife-beating. If the testicles of these men were taken out, the animal might be so far subdued that they might become harmless and useful in the ranks of labor. The penalties for crime are constantly being changed, as in the progress of civilization the individual and the character of the various forms of crime are more thoroughly understood. We respectfully suggest to the philanthropist and the legislator a careful study of the whole question.—*N. Y. Med. Times*, May, 1892.

